# Re-emergence of SARS-CoV-2 Omicron subvariant XBB.1.5: present status, treatment, and future outlook – 2023

**DOI:** 10.1097/JS9.0000000000000258

**Published:** 2023-03-13

**Authors:** Shopnil Akash, Md. Shajib Khan, Nobendu Mukerjee, Shabana Bibi

**Affiliations:** a Department of Pharmacy, Faculty of Allied Health Science, Daffodil International University, Daffodil Smart City, Ashulia, Savar, Dhaka, Bangladesh; b Department of Microbiology, West Bengal State University, Kolkata, West Bengal, India; c Department of Health Sciences, Novel Global Community Educational Foundation, Hebersham, New South Wales, Australia; d Department of Biosciences, Shifa Tameer-e-Millat University, Islamabad, Pakistan; e Yunnan Herbal Laboratory, College of Ecology and Environmental Sciences, Yunnan University, Kunming, China

Highlights

- XBB.1.5 is currently dominant in the United Kingdom.
- A newly emerging Omicron subvariant named XBB.1.5 is the most pathogenic than other variants.
- The XBB.1.5 omicron subvariant comes after the XBB and XBB.1 forms.
- The Centers for Disease Control and Prevention in the United States has described the variant as ‘fast spreading.’

*Dear Editor,*

The severe acute respiratory syndrome coronavirus, popularly known as SARS-CoV-2 (severe acute respiratory syndrome coronavirus 2), has experienced fast global dissemination during the last 3 years and has resulted in unprecedented levels of illness and death throughout the globe. The persistent appearance of new SARS-CoV-2 different variants, which have increased infectivity, pathogenicity, transmissibility, and immunological disingenuousness, has raised concerns about the efficiency of coronavirus disease 2019 (COVID-19) vaccines as well as other therapeutic drugs such as monoclonal antibodies^1^. Since its first appearance, the SARS-CoV-2 virus has undergone persistent and consistent mutation. Since the beginning of the pandemic, several distinct SARS-CoV-2 variations have been circulating. These SARS-CoV-2 variants vary somewhat from one variant to other in their capacity to produce respiratory infection during the acute phase of the virus. In the latter half of 2020, a variant with the name omicron first appeared and quickly spread around the globe.

Omicron has proved to be a challenging adversary in the fight against COVID-19. The SARS-CoV-2 Omicron variant first appeared in the year 2021 around the globe. In 2021, it made its debut in the United States and quickly spread across the country like wildfire. Since then, various omicron subvariants have developed, some of which are more pathogenic than others in evading the protection conferred by vaccination or past infection. At the beginning of 2023, a newly emerging Omicron subvariant with the name XBB.1.5 was discovered to be the most contagious strain of the virus to date. It is also thought that the number of incidents has increased due to individuals spending more time inside and hosting recent days, even though fewer people are wearing masks and doing other preventative steps. XBB.1.5 and additional Omicron subvariants, such as BQ 1.1 and BA.5, are still circulating, and specialists are working hard to understand both strains more deeply. In addition to this, they are keeping an eye on more than 300 other descendants of Omicron scattered all over the globe^2^. The XBB.1.5 Omicron subvariant comes after the XBB and XBB.1 forms and is, therefore, the third such variant. Researchers have assigned it the nickname ‘Kraken’ 3 years into the epidemic to set it apart from the ‘variant soup.’ Subvariants with an X indicate that they are the result of hybridization between two or more sublineages; in this example, BA.2.10.1 and BA.2.75.2. The WHO categorized these variants as a concern and a potential threat to public health^3^.

Despite accounting for fewer than 5% of all SARS-CoV-2 samples sequenced in the week leading up to the end of 2022, the United Kingdom Health Security Agency has said that the two varieties are most likely to become dominant in the United Kingdom. According to the agency, the variant has a mix of ‘immune escape qualities’ and a stronger ACE-2 binding affinity, which might contribute to increased transmissibility. The United Kingdom Health and Safety Executive said that while XBB.1.5 may be responsible for growth in the incidence following the current wave, it is now too early to establish this trajectory. In the meantime, the European Centre for the Prevention of Diseases has stated that there is a ‘pragmatic potential’ that XBB.1.5 would become dominant throughout the European Union and European Economic Area, which will outcome in a significant rise in the number of COVID-19 instances within the next 1–2 months. The Centers for Disease Control and Prevention in the United States has described the variant as ‘fast spreading.’ It is believed to account for around 28% of all cases in the nation (week ending 7 January 2023). ‘XBB.1.5 is projected to grow in frequency internationally and may cause a considerable proportion of confirmed cases in the nearest future’^4^.

From 22 October 2022 to 11 January 2023, a total of 5288 sequences of XBB.1.5 were detected in 38 multiple nations. The United States of America, the United Kingdom of Great Britain, Northern Ireland, and Denmark each contributed 8.1%, 82.2%, and 2.2% of these sequences, respectively. As illustrated graphically in (Figure 1)^5^.

**Figure 1:**
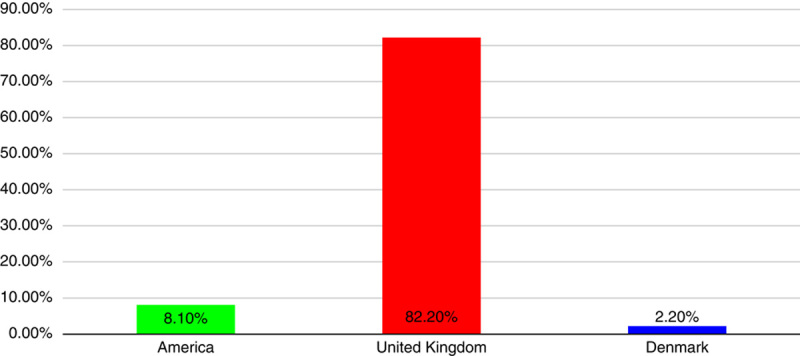
Omicron XBB.1.5 variants percentage in a different nation.

The United States Food and Drug Administration has given the majority of persons aged 6 months and older the green light to get bivalent vaccination booster doses from Pfizer-BioNTech and Moderna. These vaccines protect against the original SARS-CoV-2 virus and the Omicron BA.4 and BA.5 subvariants of the virus. In another investigation, the Centers for Disease Control and Prevention evaluated the efficiency of the bivalent vaccination against Omicron’s most recent strains, XBB and XBB.1.5, in patients who had previously been given two to four doses of the monovalent vaccine. According to the research conducted by scientists, the efficiency of the revised booster vaccine against XBB subvariants was comparable to that of BA.5 for at least the first 3 months after vaccination^2^.

In conclusion, scientists around the globe and global policymakers should be investing research funds to search for a more potent medication to fight against this pathogenic Omicron subvariant XBB.1.5. Otherwise, another pandemic may occur in the future.

## Ethics approval

Not applicable/not required. This correspondence does not require any human/animal subjects to acquire such approval.

## Sources of funding

This research did not receive any specific grant from funding agencies in the public, commercial, or not-for-profit sectors.

## Author contribution

S.A. and M.S.K.: conceptualization; S.A., M.S.K., and S.B.: writing and original draft preparation; S.B. and N.M.: writing and editing; S.B.: supervision. All authors have reviewed and approved the final version of the manuscript prior to submission.

## Conflicts of interest disclosure

The authors declare no conflicts of interest, financial or otherwise.

## Research registration unique identifying number (UIN)

1. Name of the registry: not required.
2. Unique identifying number or registration ID: not required.
3. Hyperlink to your specific registration (must be publicly accessible and will be checked): not required.

## Guarantor

I, Shopnil Akash (corresponding author), am taking full responsibility for the work and/or the conduct of the study, had access to the data, and controlled the decision to publish.

## Data availability

All data used to support the findings of this study are included in the article.
